# The prevalence and clinical significance of anemia in patients hospitalized with acute heart failure

**DOI:** 10.12688/f1000research.7872.2

**Published:** 2017-08-18

**Authors:** Attila Frigy, Zoltán Fogarasi, Ildikó Kocsis, Lehel Máthé, Előd Nagy

**Affiliations:** 1 Department of Internal Medicine IV, University of Medicine and Pharmacy Tîrgu-Mures, Târgu Mureș, Romania; 2 Department of Pharmaceutical Biochemistry, University of Medicine and Pharmacy Tîrgu-Mures, Târgu Mureș, Romania

**Keywords:** acute heart failure, prognosis, anemia

## Abstract

**Abstract: **In a cohort of patients hospitalized with acute heart failure (AHF) the prevalence of anemia and the existence of a correlation between anemia and the severity of the clinical picture were assessed.

**Methods: **50 consecutive patients (34 men, 16 women, mean age 67.5 years) hospitalized with AHF were enrolled.  Statistical analysis was performed for studying correlations between anemia and the presence/levels of diverse parameters (clinical, laboratory, echocardiographic, treatment related)  reflecting the severity and prognosis of AHF (α=0.05).

**Results:** 21 patients (14 men, 7 women, mean age 69.6 years), representing 42%, had anemia  at admission. Comparing patients with and without anemia there were no significant differences regarding age,  gender,  presence of atrial fibrillation (p=0.75), diabetes (p=1), ischemic heart disease (p=0.9), left ventricular ejection fraction (EF) (p=1), hypotension (p=0.34) and tachycardia>100 b/min at admission (p=0.75), level of eGFR (p=0.72), and need of high dose (>80 mg/day)  loop diuretic (p=0.23). However, EF showed a significant positive correlation with eGFR only in AHF patients with anemia (r=0,65, p=0.001). In a multiple regression model, EF had a significant effect on the eGFR quartiles (p=0,004).

**Conclusions:** Anemia is a frequent finding in patients hospitalized with AHF. The presence of anemia was not correlated with other factors related to AHF severity and prognosis. However, a low EF associated with low eGFR was characteristic for patients with anemia, suggesting that the decrease of renal perfusion by low cardiac output further aggravates anemia on the background of chronic kidney disease.

Anemia is relatively frequent in patients with heart failure (HF). In a population of patients with newly diagnosed HF the prevalence of anemia was 17% ^1^. The presence of anemia is related to the severity of functional class (from 9% in NYHA class I to 79% in class IV) ^2^. In acute heart failure (AHF) anemia, regardless of its etiology, could be an important extracardiac factor of decompensation; its diagnosis, evaluation and treatment being an important part of management. Also, the presence of anemia proved to be an important prognostic factor during the in-hospital and post-discharge period ^3^.

The aim of this study was to assess a cohort of patients hospitalized with AHF for (1) the prevalence of anemia and (2) the existence of correlations parameters reflecting the severity of heart failure and the grade of anemia, with special accent on decreased renal function.

## Methods

We collected data from 50 consecutive patients (34 men, 16 women, mean age 67.5 years) hospitalized with AHF (acute decompensated heart failure in 36 cases). At admission, all the patients signed the general consent form used at our institution, agreeing with anonymous data collection and usage for scientific purposes. Approval of the hospital ethical committee (permit number: 3865/01.03.2016) was obtained for data processing and publication. Exclusion criteria were: recent (<1 month) acute coronary syndrome, and advanced renal disease on hemodialysis. At admission and during hospital stay routine (part of usual care) clinical and paraclinical data were recorded in a dedicated database: demographic data, clinical diagnosis, triggering factors of decompensation, signs and symptoms at admission, ECG data, echocardiographic data, laboratory parameters at admission, and in-hospital treatment data. Anemia was defined as Hb<12 g/dL for women and Hb <13 g/dL for men. eGFR was estimated by the CKD-EPI equation.

Statistical analysis was performed with STATISTICA 5.0, using Fisher’s exact test for the comparison of discrete data, the Mann-Whitney U test for continuous parameters and the Spearman rank correlation for comparison analysis and multiple linear regression, to determine parameters influencing eGFR (α=0.05).

## Results

Patient data Please see legend.csv for a description of the data Click here for additional data file. Copyright: © 2017 Frigy A et al. 2017 Data associated with the article are available under the terms of the Creative Commons Zero "No rights reserved" data waiver (CC0 1.0 Public domain dedication).

21 patients (14 men, 7 women, mean age 69.6 years), representing 42% of the cohort, had anemia at admission. The most common form was renal anemia (10 patients), while 8 patients suffered of iron deficiency anemia. We did not find significant differences between the two groups of patients, with and without anemia, with regards to gender (p=1) and age (p=0.57). Also, there were no significant differences regarding the presence of atrial fibrillation (p=0.75), diabetes (p=1), ischemic heart disease (p=1), hypotension (systolic blood pressure <90 mmHg) at admission (p=0.34), tachycardia>100 b/min at admission (p=0.75), severe aortic stenosis (0.12), pulmonary hypertension (0.13), the level of eGFR (p=0.33), left ventricular ejection fraction (EF) (p=0.95) and need of high dose (>80 mg/day) loop diuretic (p=0.23) ( Table 1.).

**Table 1.** The comparison of diverse parameters in patients with and without anemia.

	Anemia		
Parameter	No (n=29)	Yes (n=21)	P
Age (years)	66,06 ± 2,07	69,62 ± 2,48	0,45
Gender (male/female)	20/9	14/7	1
Hemoglobin (g/L)	14,06 ± 0,19	10,76 ± 0,25	<0,001
eGFR (mL/min)	66,36 ± 4,55	60,21 ± 6,12	0,33
LV ejection fraction (%)	40 ± 3,10	40,71 ± 3,81	0,95
Ischemic heart disease	15 (51,7%)	11 (52,4%)	1
Diabetes	19 (65,5%)	13 (61,9%)	1
Atrial fibrillation	15 (52,3%)	11 (51,7%)	1
Systolic blood pressure <90 mmHg	6 (20,7%)	7 (33,3%)	0,34
Tachycardia (>100 b/min)	9 (31%)	5 (23,8%)	0,75
Severe aorta stenosis	6 (20,7%)	9 (42,8%)	0,12
Pulmonary hypertension	7 (26,9%)	11 (52,4%)	0,13
Antiaggregant treatment	2 (6,9%)	0 (0%)	0,5
Anticoagulant treatment	27 (93%)	19 (90,4%)	1
Daily ≥ 80 mg furosemid	21 (72,4%)	11 (52,4%)	0,23

We observed a significant positive correlation between eGFR and the ejection fraction (r=0,65, p=0,001) in patients with anemia, but not in those with normal hemoglobin levels (r=-0,13, p=0,48). In a multiple regression model, determining the eGFR quartiles, we found a significant effect of EF on eGFR (p=0,004).

## Discussion and conclusions

There is general agreement that anemia is a good predictor of prognosis in patients with acute and chronic HF. Anemia is associated with increased mortality, however there are conflicted data whether this is an independent predictor or reflects the progression of HF and/or is related to the presence of more frequent comorbidities ^1, 4, 5^. In the setting of AHF, anemia could also serve as a precipitating factor of decompensation.

In our cohort of patients the presence of anemia was not correlated with other factors related to AHF severity and prognosis. This fact suggests its independent role in influencing the clinical picture and prognosis. On the other hand, almost half of anemia patients suffered of chronic kidney disease, and this subgroup showed a significant association of low EF with low eGFR. Moreover, ejection fraction proved to have a significant effect on estimated glomerular filtration rate in a multiple regression model, suggesting that low EF in heart failure might cause the decrease of GFR, aggravating the chronic kidney disease and, consequently contributing to the development of renal anemia.

## Data availability

The data referenced by this article are under copyright with the following copyright statement: Copyright: © 2017 Frigy A et al.

Data associated with the article are available under the terms of the Creative Commons Zero "No rights reserved" data waiver (CC0 1.0 Public domain dedication). 

*F1000Research*: Dataset 1. Patient data, 10.5256/f1000research.7872.d122902 ^6^

## Consent

Written informed consent for publication of their clinical details was obtained from the patients.
