# Supplementary material for: The prevalence and clinical significance of anemia in patients hospitalized with acute heart failure
Source: F1000Res. 2017 Aug 18;5:1006. Originally published 2016 May 26. [Version 2] doi: 10.12688/f1000research.7872.2 (PMC5599996; doi:10.12688/f1000research.7872.2)
Supplement: Peer review history

# Peer review: Referee response for version 2

**DOI:** 10.5256/f1000research.13480.r25177

**Published:** 2017-09-13

**Authors:** José Machado

**Affiliations:** 1 Computer Science and Technology Center, University of Minho, Braga, Portugal

I have read this submission. I believe that I have an appropriate level of expertise to confirm that it is of an acceptable scientific standard.

# Peer review: Referee response for version 2

**DOI:** 10.5256/f1000research.13480.r25175

**Published:** 2017-09-11

**Authors:** Manfred Seeberger

**Affiliations:** 1 Department of Anaesthesiology and Intensive Care Medicine, Klinik Hirslanden, Zürich, Switzerland

As described by the authors, they have reconsidered their statistical analysis but the main message of the article remained the same. These changes do not address my concerns expressed regarding the first version of the article.

 "The authors have raised an interesting question. However, they need to define a more specific study hypothesis and calculate the sample size needed for analyzing that hypothesis. The current study design and sample size does not allow for drawing any reliable conclusions."

 Therefore, I am sorry to renew my concerns. I consider this study as valuable hypothesis-generating work. However, there are several reasons that should keep the authors from drawing the conclusions stated in the article:

1. The current work was not hypothesis-driven research
2. Consequently, no sample size / power calculation was made
3. Making multiple statistical analyses based on 21 patients with anemia will result in some positive findings/correlations (maybe just by chance) and many negative findings (maybe just because the sample size is dramatically inadequate).

At its current state, I cannot recommend to publish the revised version of the article. I could support publication if the findings were presented as hypothesis-generating but with the clear statement that the study lacks power for drawing valid conclusions.

I have read this submission. I believe that I have an appropriate level of expertise to state that I do not consider it to be of an acceptable scientific standard, for reasons outlined above.

# Peer review: Referee response for version 2

**DOI:** 10.5256/f1000research.13480.r25176

**Published:** 2017-08-23

**Authors:** Norbert Jost

**Affiliations:** 1 Department of Pharmacology and Pharmacotherapy, University of Szeged, Szeged, Hungary

The authors for the revised paper from "A Frigy *et al*: The prevalence and clinical significance of anaemia in patients hospitalized with acute heart failure" have answered my original concerns.

 They have now included the required data from the patients included in the cohort for the study. I suggest the acceptance of the paper.

I have read this submission. I believe that I have an appropriate level of expertise to confirm that it is of an acceptable scientific standard.

# Peer review: Referee response for version 1

**DOI:** 10.5256/f1000research.8473.r17358

**Published:** 2016-11-02

**Authors:** Norbert Jost

**Affiliations:** 1 Department of Pharmacology and Pharmacotherapy, University of Szeged, Szeged, Hungary

- **Title and Abstract**:

More or less acceptable. I would suggest adding some details about the general life quality of the patients.

- **Article content**:

Please give details about the general conditions of the patients including: i) data about other diseases (cardiac and not cardiac as well); ii) status when they arrived at the hospital and how was their status when leaving the hospital; was there post hospitalization care or not, and if yes what were the results.

- **Conclusions**:

Insufficiently short. Please supplement with information and comments about some comparative details of other studies in this field.

I have read this submission. I believe that I have an appropriate level of expertise to confirm that it is of an acceptable scientific standard, however I have significant reservations, as outlined above.

# Peer review: Referee response for version 1

**DOI:** 10.5256/f1000research.8473.r14766

**Published:** 2016-07-04

**Authors:** Manfred Seeberger

**Affiliations:** 1 Department of Anaesthesiology and Intensive Care Medicine, Klinik Hirslanden, Zürich, Switzerland

The authors have assessed the prevalence of anemia in a cohort of 50 patients hospitalized with acute heart failure (AHF), and also assessed the existence of a correlation between anemia and the severity of the clinical picture. They found anemia in 21/50 patients but no correlation of anemia with other factors related to severity and prognosis of AHF. They conclude that this finding is suggestive of an independent role of anemia in influencing the clinical picture and prognosis of AHF.

 The study by Frigy *et al *may serve as an interesting pilot study for a larger prospective study. However, the current sample size is insufficient for drawing any reliable conclusion on the prevalence of anemia in patients with AHF, and on the influence of anemia on course and outcome of AHF. Given the small sample size, it is not meaningful to perform multiple statistical analyses. And the small sample size should keep the authors from rejecting a possible correlation between anemia and other factors related to severity and prognosis of AHF. And the final conclusion remains unclear to me: why does the lack of statistical correlation between anemia and other factors related to severity and prognosis of AHF suggest an independent role of anemia in influencing prognosis of the disease? The authors have not studied prognosis and outcome at all.

 The authors need to define the study question more specifically: what is (are) the outcome(s) they are looking for in the population of patients with acute heart failure? Based on a specific study question and hypothesis, the authors need to perform a sample size calculation. It will be interesting to read the results of that adequately sized study.

 The authors have raised an interesting question. However, they need to define a more specific study hypothesis and calculate the sample size needed for analyzing that hypothesis. The current study design and sample size does not allow for drawing any reliable conclusions.

I have read this submission. I believe that I have an appropriate level of expertise to state that I do not consider it to be of an acceptable scientific standard, for reasons outlined above.

# Peer review: Referee response for version 1

**DOI:** 10.5256/f1000research.8473.r14212

**Published:** 2016-06-07

**Authors:** José Machado

**Affiliations:** 1 Computer Science and Technology Center, University of Minho, Braga, Portugal

This article addresses the prevalence of anemia amongst patients hospitalized with acute heart failure (AHF) and the existence of a correlation between anemia and the severity of the clinical picture. The manuscript is well written, but I have some concerns on certain points. Below are more specific comments by section:

- Introduction: More information about the purpose of the topic addressed would provide welcome context, i.e. the relevance of the study conducted. A bit more detail about anemia and acute heart failure would also be helpful in order to understand better the relevance of the potential correlation addressed;
- Methods: They may be some reservations concerning the data size: a small sample of data was used in order to conduct this study. On the other hand, more information regarding the methods used and how the study was specifically conducted would also be insightful;
- Discussion and conclusions: A poor discussion and conclusions are presented. Thereby, the results should be discussed in more detail, i.e. the results presented in Table 1. For instance, a more specific discussion could be done regarding the most relevant parameters presented in Table 1, i.e. parameters in patients with and without anemia.

Overall, I consider this study interesting but more information regarding certain topics seems undoubtedly needed in order to complete and clarify some crucial points addressed throughout this paper.

I have read this submission. I believe that I have an appropriate level of expertise to confirm that it is of an acceptable scientific standard, however I have significant reservations, as outlined above.
